# Broadband Antireflection and Light Extraction Enhancement in Fluorescent SiC with Nanodome Structures

**DOI:** 10.1038/srep04662

**Published:** 2014-04-11

**Authors:** Yiyu Ou, Xiaolong Zhu, Valdas Jokubavicius, Rositza Yakimova, N. Asger Mortensen, Mikael Syväjärvi, Sanshui Xiao, Haiyan Ou

**Affiliations:** 1 DTU Fotonik, Technical University of Denmark, DK-2800 Lyngby, Denmark; 2 Department of Physics, Chemistry and Biology, Linköping University, SE-58183 Linköping, Sweden

## Abstract

We demonstrate a time-efficient and low-cost approach to fabricate Si_3_N_4_ coated nanodome structures in fluorescent SiC. Nanosphere lithography is used as the nanopatterning method and SiC nanodome structures with Si_3_N_4_ coating are formed via dry etching and thin film deposition process. By using this method, a significant broadband surface antireflection and a considerable omnidirectional luminescence enhancement are obtained. The experimental observations are then supported by numerical simulations. It is believed that our fabrication method will be well suitable for large-scale production in the future.

The high-quality donor-acceptor co-doped fluorescent SiC grown by fast sublimation growth process (FSGP) has been demonstrated to be a highly efficient wavelength-conversion material for white light-emitting diodes (LEDs)^1^,^2^. Compared with the normal wavelength-conversion material like phosphors, fluorescent SiC is much superior in terms of high color rendering index and long lifetime. Also, SiC has an excellent thermal conductivity and a small lattice mismatch with GaN (critical material for blue LEDs), thus making itself a well-established substrate material for nitride growth. However, SiC has a rather high refractive index (2.65 at 580 nm)^3^, indicating that most of the emitted light is confined in the material and can not escape into air due to the total internal reflection, i.e. a very low extraction efficiency. To enhance the light extraction efficiency of the SiC-based white LED, different nanopatterning approaches (e-beam lithography, self-assembled metal nanoparticles etc.) have been developed to fabricate an antireflection structure (ARS) array on a SiC surface^4^,^5^,^6^. Among these methods, a metal template is always needed to resist a strong etching power in order to break the strong atomic binding energy of the SiC. However, metals are routinely excluded by a lot of semiconductor processing facilities and they are rather expensive when considering any future mass production. Therefore we propose a metal-free method, i.e, using self-assembled polystyrene (PS) nanospheres as a template, to fabricate nanodome structures on nitrogen-boron co-doped fluorescent 6H-SiC by using reactive-ion etching (RIE) process. Compared to the commonly used e-beam lithography and nanoimprint lithography processes, nanosphere lithography is a time-saving and cost-efficient nanopatterning technique for subsequent etching approaches and it is also a promising way for large-scale production^7^,^8^,^9^,^10^,^11^,^12^,^13^. Moreover, self-assembly nanospheres with different material and dimension choices are commercially available, thus providing an attractive platform in various applications.

In the paper, B and N co-doped fluorescent 6H-SiC samples (200 μm grown by FSGP) were processed by nanosphere lithography and reactive ion etching (RIE). The wavelength-scale nanodome structures formed at the surface suppress the average surface reflection over the whole visible spectral range and enhance the omnidirectional luminescence. The antireflection and luminescence enhancement performance of the nanodome structures are further enhanced by depositing a thin Si_3_N_4_ coating on top of the nanodome structures. To the best of our knowledge, this is the first report using PS nanospheres as template to form nanodome structures on SiC.

## Results

**Figure 1:**
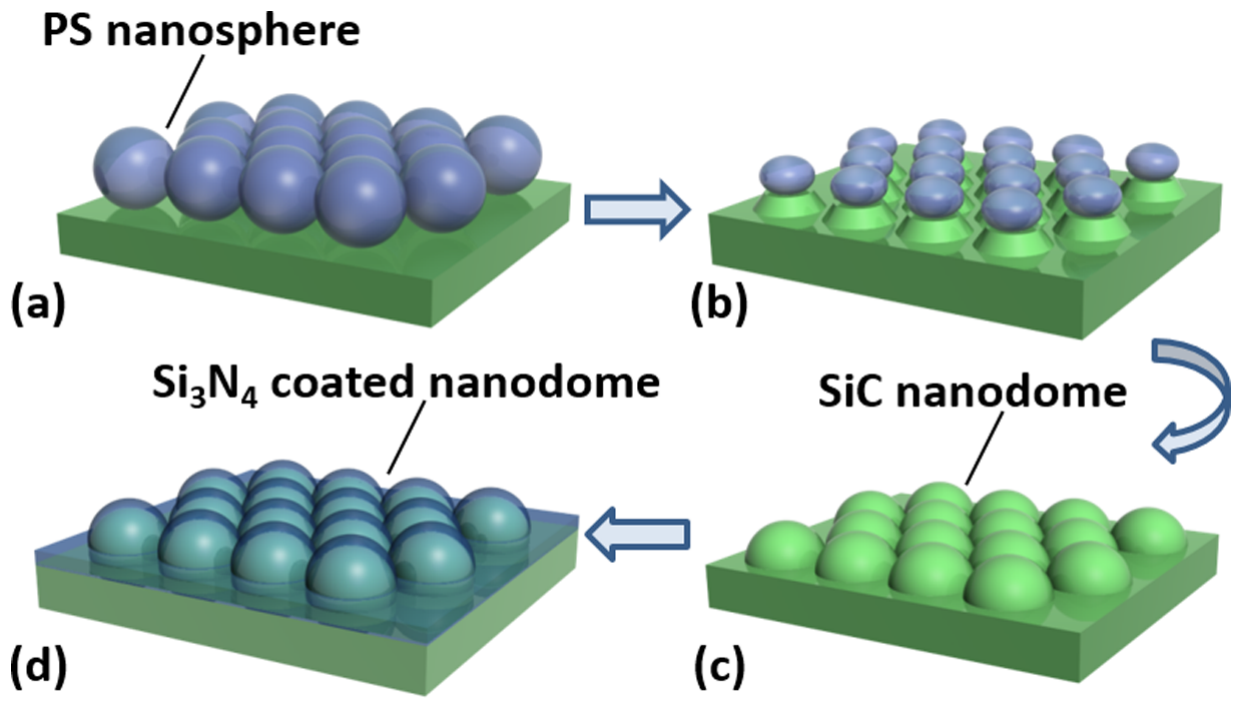
Schematic diagram showing the detailed fabrication process of Si_3_N_4_ coated nanodome structures on fluorescent SiC samples. (a) Formation of self-assembled polystyrene monolayer nanospheres as etching template, (b) dry etching process by RIE with SF_6_ and O_2_ gases, (c) formation of the nanodome structures on fluorescent SiC samples and, (d) Si_3_N_4_ film deposition on SiC nanodome by PECVD.

A schematic drawing of the detailed fabrication process of the nanodome structures is illustrated in Fig. 1. Firstly, a monolayer hexagonal-close-packed array of PS nanospheres with a diameter of 600 nm (size dispersion of 1%) was formed on a pre-treated SiC sample surface by a self-assembly method (see Fig. 1(a))^14^,^15^,^16^. Subsequently, the SiC sample was subjected to RIE (STS cluster system C010) for pattern transfer, where the PS nanospheres monolayer serves as an etching template (see Fig. 1(b)). Due to the isotropic nature of the chemical etching from the plasma gases and directional bombardment of accelerated ions from the physical etching in RIE, the PS nanospheres were etched away in an anisotropic way. Consequently, the nanospheres became thinner and their transverse diameters also decreased gradually. The exposed area of the underlying SiC substrate then increased. After 5 minutes etching, the nanospheres are consumed completely and SiC nanodome structures are achieved by using the PS nanospheres (shrinking during the etching) as the unique template (see Fig. 1(c)). In addition, a 57 nm thick Si_3_N_4_ coating with an intermediate refractive index (n = 2.0) between the value of air (n = 1) and 6H-SiC (n = 2.65) was deposited on top of the nanodome structures by plasma-enhanced chemical vapor deposition (PECVD) to further enhance the extraction efficiency.

**Figure 2:**
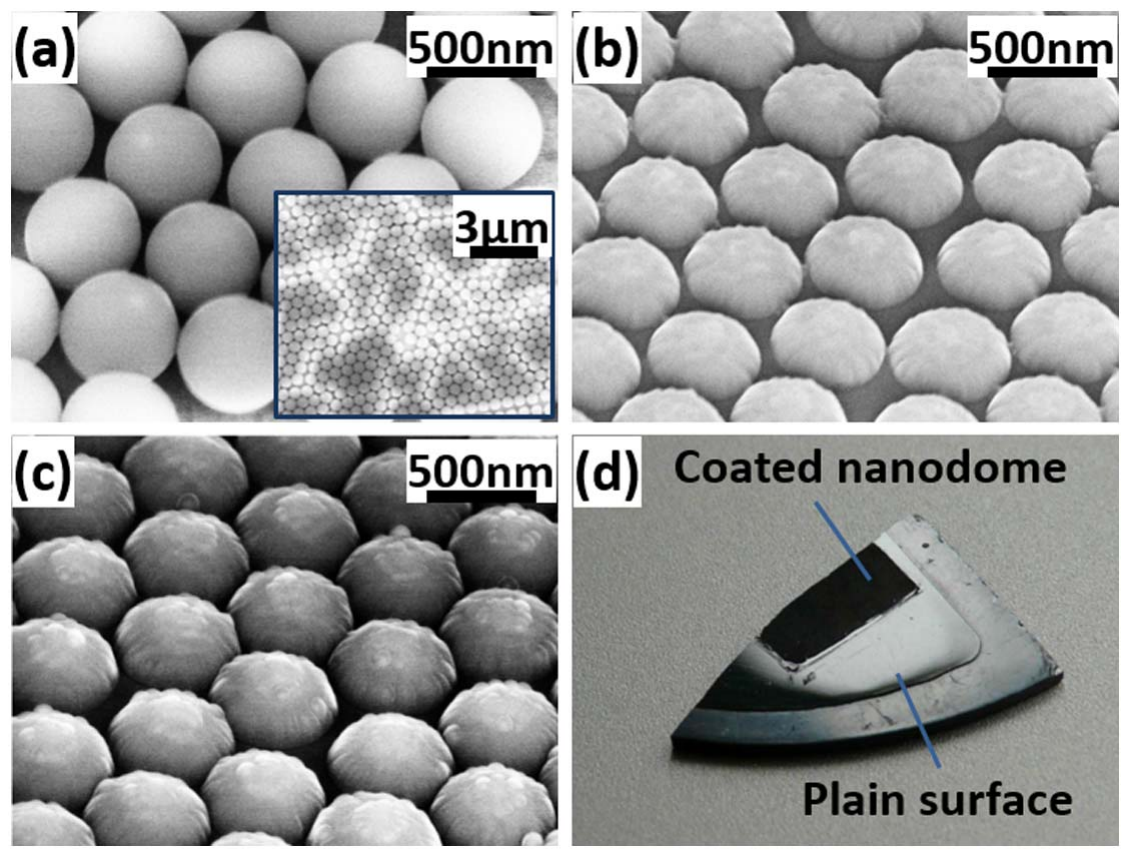
Oblique-view SEM images of the (a) monolayer hexagonal-close-packed polystyrene nanospheres (inset is a large-scale view), (b) fabricated SiC nanodome structures, and (c) Si3N4 coated nanodome structures respectively. (d) A photograph of SiC sample with a partially plain surface (lower right part) and partially covered by the coated nanodome structures (upper left part).

Oblique-view SEM images of the close-packed monolayer PS nanospheres, SiC nanodome structures (ND-SiC), and Si_3_N_4_ coated nanodome structures (CND-SiC) are demonstrated in Figs. 2(a)–2(c), respectively. A large view SEM image is inset in Fig. 2(a) to show the scalability of this method. The nanodome structure has a structure height of around 200 nm (measured directly from cross-section SEM image) with a semi-spherical shape and smooth surface. Meanwhile, the aspect ratio of the nanodome structure is low which is a desired property for further Si_3_N_4_ film deposition. On the other hand, in Fig. 2(c), the Si_3_N_4_ coated nanodome exhibits a larger structure height and a relatively rough surface. Fig. 2(d) shows a photograph of the SiC sample with a partially plain surface (plain-SiC) and a partially coated nanodome covered surface(coated nanodome). The surface color of the sample turns from bright grey to black after surface patterning, indicating a significant suppression of surface reflection.

**Figure 3:**
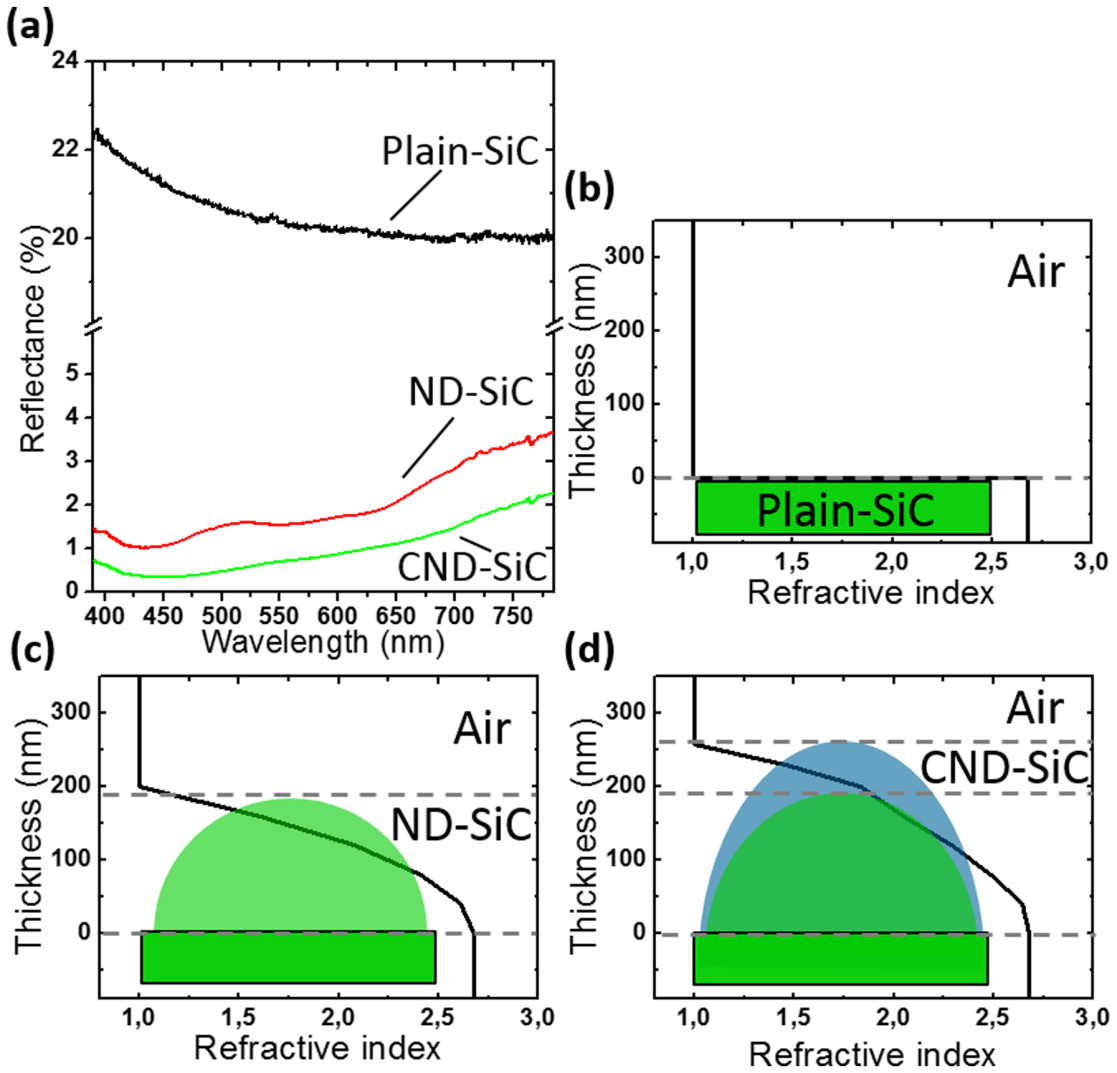
(a) Measured optical reflectance of plain-SiC, ND-SiC, and CND-SiC. (b–d) Schematic illustrations and calculated effective refractive index profiles of plain-SiC, ND-SiC, and CND-SiC respectively.

The measured reflectance spectra in a wavelength range of 390–785 nm are shown in Fig. 3(a). In the measured spectral range, the average reflectance of plain-SiC is around 20.5%. Due to the graded refractive index profile of ND-SiC, the average reflectance is significantly suppressed to 2.0%, while the reflectance is below 4% throughout the whole wavelength range. A lower average reflectance of 0.99% is obtained for the CND-SiC sample. This is because the Si_3_N_4_ coating further relieves the refractive index difference between the air/SiC interfaces. The above experimental observations are explained by the effective refractive index (n_E_) profiles of the interfaces between air and different SiC samples, as shown in Figs. 3(b) to 3(d). The n_E_ profile could be approximated by n_E_ = f n_1_ + (1 − f) n_2_^17^, where n_1_ and n_2_ are the refractive indices of fluorescent SiC (n = 2.65) and air (n = 1) respectively, and f is the filling factor weighted by the structure volume at different structure heights. As shown in Fig. 3(b), the refractive index changes abruptly from 2.65 to 1 across the plain interface, which causes large internal/surface reflection. For the ND-SiC, the diameter of the dome structure shrinks gradually from the bottom to the top, thus resulting in a graded transition of the n_E_ (see Fig. 3(c)). As a result, nanodome structures are anticipated to achieve considerable reflection suppression over a broadband spectral range. Compared with ND-SiC, CND-SiC with a Si_3_N_4_ coating which has an intermediate refractive index (n = 2.0) can further relieve the refractive index difference in a more smooth way (see Fig. 3(d)), thus an even lower surface reflection could be expected. These expectations are supported by our experimental results shown in Fig. 3(a). The choice of Si_3_N_4_ coating is just for the proof-of-concept. Further suppression of the antireflection could be achieved by carefully engineering the refractive index and thickness of the coating.

**Figure 4:**
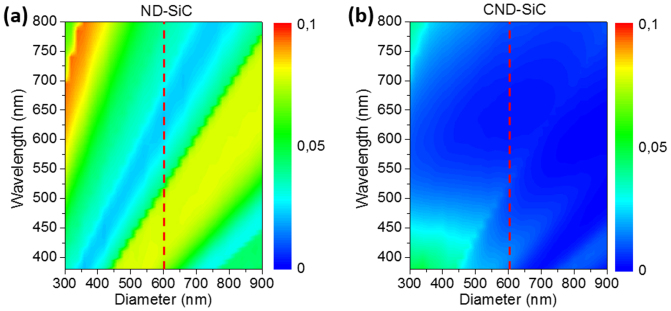
Simulated reflectance of the SiC sample as a function of wavelength and nanosphere diameter for (a) ND-SiC and (b) CND-SiC.

**Figure 5:**
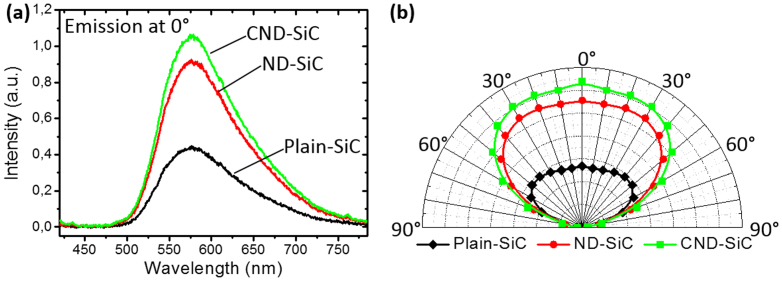
(a) Photoluminescence spectra at emission angle of 0° and (b) angular emission profiles of plain-SiC, ND-SiC, and CND-SiC respectively.

In order to estimate the influence of the sphere diameter on the optical antireflection enhancement, we illustrate in Fig. 4 the total optical reflectance of ND-SiC and CND-SiC as a function of the sphere diameter calculated by a rigorous coupled-wave analysis (RCWA) model. The sphere diameter varies between 300 and 900 nm. In general, CND-SiC has even lower reflectance than ND-SiC. We again observe a fairly low reflectance for both ND-SiC and CND-SiC with a diameter of 600 nm as we used (the dash line in the figure), especially in the wavelength region of 550–650 nm where the luminescence peak of SiC is located. As seen from Fig. 5(a), the fluorescent SiC emits at peak wavelength of 575 nm, therefore 600 nm diameter nanospheres were chosen as the template with the guidance of the simulation results in Fig. 4. Moreover, by tuning the sphere size, the spectral response to the antireflection can be adjusted accordingly. Therefore, such feature can be applied for different wavelength purpose.

For the two orthogonal polarizations, both the luminescence spectrum at the emission angle normal to the sample surface and the angle-resolved photoluminescence are quite similar to each other. So results for only one polarization are shown in Fig. 5. Fig. 5(a) shows that the fluorescent SiC sample exhibits a broad luminescence peaked at 575 nm and the luminescence is enhanced after surface nanostructuring. Compared to plain-SiC, the ND-SiC demonstrates a 107% luminescence enhancement at the emission angle normal to the sample surface. Meanwhile, the luminescence of the CND-SiC is enhanced significantly by 138%. The observed photoluminescence enhancement results are in a good consistency with the experimental observations from the surface reflectance suppression measurements.

Angular integral emission intensity profiles from angle-resolved photoluminescence measurements are also illustrated in Fig. 5(b) for plain-SiC, ND-SiC, and CND-SiC samples. A broadband extraction enhancement is obtained for both ND-SiC and CND-SiC samples at all the tested angles. Especially, a large enhancement over 50% is obtained at a large emission angle up to 60° for both samples.

## Discussion

Compared to periodic nanocone structures in Ref. 5, nanodome structures in this paper has very close luminescence enhancement effect. The merit of these nanodome structures is they have much lower aspect ratio than nanocone structures, thus it could benefit from a capping layer with medium refractive index (like Si_3_N_4_) to further enhance the extraction efficiency.

In conclusion, we have demonstrated a novel approach to fabricate nanodome structures in fluorescent SiC. This attractive approach relies on a RIE process with self-assembled monolayer PS nanospheres as a template and a thin film deposition process. Compared with a plain sample, the coated nanodome structures present a graded transition of the effective refractive index from the structure bottom to the surface which leads to a significant reflectance reduction from 20.5% to 0.99% in the spectral range of 390–785 nm. Numerical simulations were implemented to further support the experimental results. A considerable luminescence enhancement of 138% is also achieved and large luminescence enhancement can be maintained in a large emission angle range. Related applications such as solar cells and optical detectors could also benefit from the excellent antireflection performance by using this low-cost and scalable fabrication method.

## Methods

Optimized RIE conditions were applied with a R.F. power of 100 W, chamber pressure of 30 mT, and a mixture of SF_6_ and O_2_ gases (24 and 6 sccm). The Si_3_N_4_ thin film was deposited by PECVD (STS cluster system C010). The thickness of Si_3_N_4_ thin film is measured on a dummy wafer, which has a plain surface and is deposited at the same time as the nanodome sample, by using filmtek (Filmtek 4000 Spectrometer). The etched profile and surface morphology of the processed SiC samples were then observed by a scanning electron microscope (SEM LEO 1550, Carl Zeiss). Surface reflectance measurements of different SiC samples were carried out by using a goniometer system (Instrument Systems, GON360). A halogen lamp as a broadband light source was connected to the transmitter arm of the goniometer and the receiver arm was connected to an optical spectrometer (Instrument Systems, CAS140B). SiC samples were mounted on the sample stage and the reflection spectra were measured with an incidence angle of 8° with respect to the surface-normal direction. Angle-resolved photoluminescence measurements were carried out by using the same goniometer. A 377 nm diode laser was connected to the transmitter arm as the excitation light source and the SiC sample was optically excited from its back side at room temperature. The detection angle of the receiver arm was varied from 0° (surface-normal direction) to 85° with a step of 10° and the corresponding photoluminescence spectra at two orthogonal polarizations were then acquired.

## Author Contributions

Y.O. and H.O. wrote the main manuscript text. Y.O. prepared figures 1–3, 5 and X.Z. prepared Fig.4. V.J. grew the f-SiC samples. X.Z. deposited nanosphere on top of f-SiC, and Y.O. did the sample processing and characterization. R.Y., M.S., S.X., N.M. and H.O. supervised the study. All authors reviewed the manuscript.
